# Why and How to Switch to Genomic Selection: Lessons From Plant and Animal Breeding Experience

**DOI:** 10.3389/fgene.2021.629737

**Published:** 2021-07-09

**Authors:** R2D2 Consortium, Aline Fugeray-Scarbel, Catherine Bastien, Mathilde Dupont-Nivet, Stéphane Lemarié

**Affiliations:** ^1^Université Grenoble Alpes, INRAE, CNRS, Grenoble INP, GAEL, Grenoble, France; ^2^INRAE, BioForA, Orléans, France; ^3^Université Paris-Saclay, INRAE, AgroParisTech, GABI, Jouy-en-Josas, France

**Keywords:** prediction accuracy, duration of breeding cycle, selection intensity, breeder’s equation, breeding organization, breeding program, selection costs, Mendelian sampling

## Abstract

The present study is a transversal analysis of the interest in genomic selection for plant and animal species. It focuses on the arguments that may convince breeders to switch to genomic selection. The arguments are classified into three different “bricks.” The first brick considers the addition of genotyping to improve the accuracy of the prediction of breeding values. The second consists of saving costs and/or shortening the breeding cycle by replacing all or a portion of the phenotyping effort with genotyping. The third concerns population management to improve the choice of parents to either optimize crossbreeding or maintain genetic diversity. We analyse the relevance of these different bricks for a wide range of animal and plant species and sought to explain the differences between species according to their biological specificities and the organization of breeding programs.

## Introduction

Genomic selection (GS) was first introduced by Lande and Thompson (2000) and popularized by Meuwissen et al. (2001). This method is based on the use of high-density single nucleotide polymorphisms (SNP) genotyping to predict breeding values. The development of a genomic breeding program requires two steps: (1) in a reference population, individuals are genotyped and phenotyped, and a statistical model is then built to estimate SNP effects on phenotypes and develop corresponding prediction equations; and (2) new candidates for selection may or may not be phenotyped but are always genotyped, and their breeding values are predicted using prediction equations and phenotypes when available.

Genomic selection was first implemented in dairy cattle. GS methods have been developed quickly due to the possibility of estimating the breeding value of bulls for milk production early and precisely through genomic prediction equations rather than later through a costly progeny test (Schaeffer, 2006; Hayes et al., 2009; Venot et al., 2016; Wiggans et al., 2017). However, GS is based on generic methods and technology and can potentially be implemented for the breeding of any plant or animal species, as long as breeding aims at improving polygenic traits. Indeed, selection for many plant species addresses polygenic traits. This is the case for field crop species with yield and other traits (e.g., adaptation to climate, size). For vegetables, major traits are controlled by numerous quantitative trait loci (QTL) [see Zhao et al. (2019) for quality traits and Bai et al. (2018) for biotic and abiotic stresses]. However, part of the traits of interest for plants are monogenic: color or form of the harvested organ (vegetable), some disease resistances (crop or vegetable). GS is not relevant to improve these monogenic traits.

Depending on the species, the organization of the breeding programs and the constraints on breeding may be very different. As a consequence, GS can be implemented differently to relax these species and breeding program constraints. For e.g., annual crops have short life cycles, but the phenotyping of these crops to cover environmental variability between locations and years and control for genotype-by-environment interactions is very costly. The life cycle is also short for some animal species, such as poultry or pigs, but a major constraint in these species is the lethal phenotyping of specific traits of economic interest. Thus, these traits are evaluated from sibs performance. Finally, the best strategy for implementing GS for a given species can change over time according to the evolution of technology, knowledge, and breeding goals.

Many articles have explored the advantages and drawbacks of GS, focusing on a single species or a limited set of related species (Meuwissen et al., 2013; Heslot et al., 2015; Lin et al., 2016). These reports are often prospective, being produced before the actual implementation of GS, rather than retrospective. Additionally, they often include all the advantages independent of their importance. Thus, they do not provide reasoning that would ultimately or potentially convinces stakeholders to switch to GS. Finally, with the exception of the work by Jonas and de Koning (2013) and Hickey et al. (2017), to our knowledge, there has been no joint analysis of the arguments for GS in plant and animal species.

In this article, our aim is to propose a common framework for analysing the multiple arguments for implementing GS in a large range of plant and animal species for the breeding of polygenic traits. Our first aim is to synthesize the basic arguments justifying the potential interest in using genomic information in any plant or animal breeding program. These arguments are defined as complementary basic bricks that can possibly be combined for the breeding of any species of interest in agriculture. As far as possible, we focus on the tipping point that is specific to each species and not on a whole range of possible applications of GS in the future. Before presenting these bricks and their importance, we first briefly review key features of the biological specificities and organization of breeding programs for the various species.

This study is based on an analysis of the literature and on multiple exchanges within the expert group R2D2 supported by the INRAE SELGEN metaprogram^1^. This group included French researchers in the field of GS (including geneticists as well as economists) in a wide range of commercially selected animal and plant species (dairy and beef cattle, dairy and meat sheep, dairy goats, pigs, horses, laying hens, broilers, fish, wheat, rice, maize, peas, forage crops, forest trees, fruit trees, oil palm trees, tomato, and grapevine). Members of this group have met twice a year since 2012, with part of each meeting being devoted to the discussion of GS strategies for each species and the comparison of these strategies among species.

## Materials and Methods

### Biological and Breeding Organization Specificities

Before analyzing the different arguments for the interest in GS implementation, it is important to review the major differences in breeding organizations and constraints among species. These differences are due to the biological specificities of each species and to the different types of selection products that are commercialized (e.g., seed, artificial insemination semen, young plants, broodstock, and juveniles). These selection products are generally such that they enable the wide diffusion of genetic gains at a rather low cost, according to the known biological specificities of each species and the available technologies.

The first important characteristic of the breeding organization is the duration of the breeding cycle. A breeding cycle is initiated by crosses/mating between selected parents, and the duration of this cycle is the time between two initial crosses. This duration does not take into account the duration of commercial development, which may necessitate extra generations for multiplying selected plants or animals. For various reasons, the breeding cycle duration varies from 1 year to one or several decades. Here, we review the main constraints determining the duration of the breeding cycle.

The breeding cycle duration is first constrained by the age at sexual maturity, which determines the shortest possible length of time between two successive generations and is incompressible due to biological limits. For animals and trees, a breeding cycle corresponds to one generation. For crops and vegetables, the breeding cycle typically encompasses several generations to produce enough seeds for repeated field trials at multiple locations. Additionally, when doubled haploids cannot be produced at a reasonable price (e.g., peas), several generations are necessary to obtain homozygous genotypes, which is usually a requirement for obtaining intellectual property rights and variety registration.

The duration of the breeding cycle can also be constrained by the way in which phenotyping is implemented. For example, cycles are longer when offspring evaluation is required to select males on the basis of female traits (e.g., male selection for dairy production in ruminants) or when backward selection is necessary (e.g., in forest tree breeding). This is also the case when selection is based on values obtained from hybrid combination (e.g., in maize). Cycles can also be long when some traits can only be phenotyped at late stages (e.g., wood production for forest trees, persistence of forage crops, and horse sport performances).

Finally, taking into account all the constraints described above, the duration of the breeding cycle in animal species before the implementation of GS is highly variable, ranging from 1 year for broilers to 2 years for laying hens and pigs, 2–4 years in most fish species, 4–5 years in small ruminants, 5–6 years for cattle and up to 10–11 years for horses. In plant species, the duration of the breeding cycle is always long, ranging from 8 to 10 years for wheat and ray grass (or 5 years, when doubled haploids are used) to the longest durations of up to 20 years for a selection cycle in fruit or forest tree.

The second important characteristic of breeding programs concerns genetic evaluation. Two factors, evaluation cost and accuracy, are to some extent interrelated because accuracy can be improved by spending more on the evaluation of each candidate. However, accuracy is primarily dependent on the traits to be evaluated to meet the needs of farmers, stakeholders, and society. Some traits are difficult to evaluate precisely because they exhibit low heritability per se (e.g., pig prolificacy), high genetic × environment interactions, or are difficult to measure (e.g., resistance to Aphanomyces fungi in pea, feed efficiency in fish, sensory quality in tomato, and resilience to drought in forest trees), or correspond to lethal traits that can only be measured in sibs (e.g., meat quality or animal disease resistance). The evaluation cost is related to the traits that need to be evaluated but is also determined by the species involved (linked to size, prolificacy, and age at sexual maturity) and the possibility of controlling environmental effects: this cost is very high for bulls or trees, quite cheap for fish or poultry and intermediate for crops.

### Genomic Selection Bricks

According to the analysis of the evolution of breeding programs in numerous animal and plant species, we hypothesize that the switch from classical selection to GS occurred first because of one main, simple argument. We describe possibilities that are sufficient to cover all situations and analyse why and how GS has been or could be implemented for different species. Each possibility, which we refer to as a brick, is a specific argument for carrying out GS. Each brick must not be considered as a detailed reality since genomic breeding programs generally combine several bricks. Bricks must be considered simple alternatives that are first implemented and subsequently convince stakeholders to switch to GS. In any scenario considered here, we assume that a reference population is available and composed of individuals phenotyped and genotyped with a technology providing enough SNPs.

Three bricks are proposed. These bricks correspond to the decision of the breeder when he defines the breeding scheme. Two bricks directly concern the improvement of traits: adding genotyping while retaining phenotyping in order to increase accuracy (brick A) or replacing all or part of the phenotyping effort with genotyping in order to reduce the cost of evaluating candidates (brick B). The last brick (brick C) aims at improving the choice of parents to either optimize crossbreeding or preserve genetic diversity. Brick C concerns both the improvement of traits and population management.

Each of the three bricks impacts some of the parameters of the breeder’s equation, which describes the expected genetic gain at a given selection cycle for recurrent selection of polygenic traits:

$$\Delta \,G=\frac{i\cdot r\cdot \sigma_{G}}{T}$$

Where Δ*G* is the genetic gain per year, *i* is the intensity of selection, *r* is the accuracy of selection, σ_*G*_ is the genetic standard deviation, and *T* is the duration of the breeding cycle. Thus, to improve Δ*G*, there must be an increase in *i*, *r*, or σ_*G*_, or a decrease in *T*. The effects of the bricks on the parameters of the breeder’s equation are summarized in Table 1. Each brick has several positive impacts, which are denoted by “+” for direct impact and “(+)” for indirect impact. These impacts are explained in more detail below.

**TABLE 1:** Impact of each brick on the parameters of the breeder’s equation.

Brick/parameter of the breeder’s equation	*i*	*r*	σ_*G*_	*1/T*
(A) Add genotyping to increase selection accuracy	(+)	+		(+)
(B) Replace all or part of the phenotyping effort with genotyping	+			+
(C) Improve the choice of parents to optimize crossbreeding or preserve genetic diversity		+	+	

**i*, intensity of selection; *r*, accuracy of selection; σ_*G*_, genetic standard deviation; *T*, duration of the breeding cycle. Impacts are denoted by “+” for direct impact and “(+)” for indirect impact.*

#### Brick A: Adding Genotyping to Increase Selection Accuracy, *r*

In this brick, all candidates for selection or their relatives (for the evaluation of lethal or sex-specific traits) are still phenotyped, but genotyping is added to improve the accuracy of the estimated breeding values of candidates for selection. Hence, additional costs related to genotyping are incurred to increase selection accuracy. This brick is interesting in two main situations.

The first situation corresponds to cases where the direct phenotyping of candidates for selection is not possible (for e.g., in the case of lethality related to disease resistance or sex-linked traits). In these cases, information from related individuals is traditionally used to predict breeding values. In practice, this situation can occur if full or half sibs are phenotyped in a collateral test or if candidates are chosen on the basis of pedigree information. Without genomic information, all individuals of the same family exhibit the same estimated breeding value. However, because of Mendelian sampling, all individuals do not have the same genotype, and they differ in their “true” breeding values. Thus, in conventional breeding programs, where candidates are evaluated from relatives, genetic gains are limited because it is not possible to exploit within-family genetic variability. GS allows us to discriminate between full sibs on the basis of the estimation of molecular information, which improves selection accuracy.

Brick A is interesting in a second type of situation in which traits are complicated to measure, and/or a large amount of data is required to accurately predict breeding values. This is the case for traits with low heritability or those characterized by high genotype by environment interactions, leading to imprecise breeding values or costly phenotyping designs. In this case, genomic prediction is of particular interest to increase the accuracy of breeding values. In this brick, the phenotyping of candidates (and possibly their relatives) is maintained for all individuals but can be reduced to some extent (e.g., fewer repetitions, earlier measurements, and fewer environments) to lower costs and/or decrease the difficulty of phenotyping.

The main expected effect of brick A is the improvement of the accuracy, ***r***, due to the genomic information. Therefore, it may be relevant for the breeder to decrease the precision of phenotypic measurements (which are still collected in all candidates), leading to more candidates and, consequently, higher intensity of selection, ***i***. In some cases, the reduction of phenotyping effort can be achieved through earlier phenotyping, which may affect the duration of the breeding cycle, ***T***.

#### Brick B: Replace All or Part of the Phenotyping Effort With Genotyping to Increase *i* or Decrease *T*

Another advantage of introducing genomic information is that large decreases in the phenotyping effort can be achieved by removing phenotyping for all or a large portion of selection candidates. The impacts of this brick are a decrease in phenotyping costs and the possibility of performing selection very early. It relies on the assumption that genomic accuracy is fairly good compared to phenotypic prediction. One must emphasize that the reduction of phenotyping concerns the candidates and that phenotyping effort should be maintained in the reference population to maintain accurate prediction equations. Two versions of this brick are possible depending on whether all or part of the phenotyping effort is replaced by genotyping.

In the first situation, phenotyping is fully replaced by genotyping. In this case, the duration of the breeding cycle decreases because genotyping enables early selection without waiting for phenotypes to be measured. For example, it is possible to remove progeny testing for species or traits for which the progeny testing procedure is lengthy and considerably exceeds the age at sexual maturity. However, the gain in *T* is constrained by the age at sexual maturity (*T* cannot be lower than this age) and/or by the need to include several generations in one selection cycle, for example, to produce enough seeds for field trials in crops.

In the second situation, genomic prediction is used to conduct pre-screening and eliminate the worst candidates. The pre-screened candidates are then selected using phenotypes and genotypes together. More candidates for selection are then produced, leading to an increase in the selection intensity, ***i***. It should be observed that the combined use of phenotypes and genotypes of the pre-screened candidates corresponds to brick A, leading to a possible improvement of the accuracy *r*. Hence, this case with pre-screening is one particular case leading to the combination of two bricks: the prime objective corresponds to brick B, but genomic prediction used for pre-screening enables also to implement brick A without extra cost.

The expected impact of this brick is mainly a decrease in ***T*** or increase in ***i***. The reason for the decreasing ***T*** in this case is quite different from that under brick A. Under brick A, ***T*** is decreased because it becomes possible to perform phenotyping earlier, but all candidates are still phenotyped. Under brick B, ***T*** is decreased because the first objective is to remove or greatly limit phenotyping. Brick B can potentially have negative effects on accuracy, ***r***. The change in ***r*** needs to be estimated and balanced with the gains in ***i*** and ***T*** to evaluate the interest in brick B.

#### Brick C: Improve the Choice of Parents to Either Optimize Crossbreeding or Preserve Genetic Diversity

This brick mainly concerns the management of genetic diversity. GS can be used to help choose which parents to cross. Under classical selection, because of budget constraints, only a fraction of the possible crosses can be performed. Two versions of this brick are possible depending on whether the objective is the short-term genetic progress or long-term preservation of genetic diversity and maximization of recombination.

In the first case, especially in crops, the selected candidates come from abundant and diverse progeny from a cross between two parents. Breeders have to choose the parents that are crossed at the beginning of a new breeding cycle. To obtain the best possible progeny, the parents must not only present the highest breeding values but must also show a good level of complementarity. The choice of complementary parents can be made on the basis of phenotypic information (i.e., choosing parents with complementary performance), but this can be improved significantly by using genomic information.

In the second case, genotypes provide access to genomic relationships, which are more precise than classical pedigree relationships and, thus, enable better management of genetic variability based on knowledge of the realized kinship between individuals.

The expected impact of this brick is mainly an increase in σ_*G*_. In the short term, the objective is to better exploit current genetic diversity. In the long term, the objective is to maintain genetic diversity and the level of σ_*G*_ after several breeding cycles. This expected gain from brick C is of potential interest for all species.

For each brick and for each animal or plant species, we determined the brick that was initially used to implement GS or could be used initially when GS has not yet been implemented. This analysis is based on the literature and discussion within the R2D2 expert group. The literature includes various articles reporting how GS has been implemented, articles reporting the efficiency of some strategies based on simulations or specific experiments, and finally, articles in which experts discuss how GS could be implemented for some species.

## Results

For brick A, the first situation we considered is when the direct phenotyping of candidates for selection is not possible. It occurs in animal species such as fish and pigs, in which the evaluation of sib performance is largely applied and allows to increase selection accuracy (Sonesson and Meuwissen, 2009; Tribout, 2011; Robledo et al., 2018). A related approach could be interesting for forest trees (Plomion et al., 2016) or forage plants when polycrosses (crosses using a mixture of pollens) are implemented: GS allows the redrawing of pedigrees, on the basis of data from fathers in particular, thus increasing the accuracy of selection (Riday, 2011; Vidal et al., 2017).

Brick A is also interesting in a second type of situation in which traits are complicated to measure, and/or a large amount of data is required to accurately predict breeding values. The interest in GS to improve selection accuracy for traits with low heritability is frequently highlighted for animal species such as beef cattle (Garrick, 2011), poultry (Wolc et al., 2016), or pigs (Tribout, 2011). For the last species, GS has actually been implemented in Landrace pigs in France since 2016 for the selection of reproduction traits, which exhibit low heritability (Bouquet et al., 2017). Bouquet et al. (2017) showed that, even if the accuracy remains low for these traits with low heritability, the accuracy is much better than with pedigree evaluation. The use of GS to better select for low-heritability traits is also reported in plant breeding (Endelman et al., 2014; Michel et al., 2017, for baking ability for winter wheat). Nevertheless, the literature on plant breeding more often highlights the interest in GS to control for genetic by environment interactions (Crossa et al., 2017; Rutkoski et al., 2017). As considerable variations in the environment may occur from 1 year to the next, genomic prediction based on data from several years may help control for this interaction (He et al., 2016). GS is also used to improve predictions when the objective is to develop hybrid varieties in plants (e.g., maize) or to cross purebred parental lines in animals (e.g., poultry or pigs). GS can be used to predict the crossing ability in cases where it has not yet been evaluated (e.g., in pigs, Tribout, 2011; Samorè and Fontanesi, 2016; Tusell et al., 2016) or to improve such predictions by predicting the specific combining abilities (SCA) for pairs of individuals, in addition to their general combining ability (GCA) for crossing (Bernardo, 1994; Zhao et al., 2015; Kadam and Lorenz, 2018; 28–31; Seye et al., 2020).

Globally, in the history of GS, the brick A was not the first to be implemented because it generally leads to an increase in the total cost of breeding. Thus, the adoption of this brick took more time, as it was necessary to carefully estimate the benefit of GS and convince breeders of its usefulness. Nevertheless, the interest in this brick increases as the cost of genotyping decreases.

Concerning the brick B, the strategy where phenotyping is fully replaced by genotyping has been used or is planned to be implemented in species with long breeding cycles, such as dairy cattle and trees. In dairy cattle, the introduction of GS in France in 2009 led to changes in the breeding program organization and greatly decreased *T* from 5 years to 2–2.5 years for the male pathway. Indeed, it allowed the reduction of progeny testing, as the breeding values of young bulls could be estimated at birth, without waiting to observe their daughters’ performance (Schaeffer, 2006; Hayes et al., 2009; Venot et al., 2016). This leads to a considerable decrease in cost. Because of these obvious advantages, this scenario represented the first case of the implementation of GS at a large scale in many countries. Because of decreasing the duration of the breeding cycle, this strategy is also of interest in the case of forage plants (Annicchiarico et al., 2015; Lin et al., 2016). For crop species such as wheat, variety development requires several generations of selfing to multiply seeds. One possible strategy is to replace some normal breeding cycle(s) with short breeding cycle(s) based only on GS (Beyene et al., 2015; Longin et al., 2015; Cericola et al., 2018; Pembleton et al., 2018). Trees (forest trees and fruit trees) are also species for which the duration of the selection cycle is very long and could be drastically decreased via the implementation of brick B (Kumar et al., 2012; Biscarini et al., 2017; Grattapaglia, 2017; Gorjanc et al., 2018). Decreasing the duration of the breeding cycle is also an interesting option for selection of roosters in laying hens (Le Roy et al., 2014).

The other option of the brick B where genomic prediction is used to conduct pre-screening has been adopted in dairy sheep and goats, in which the selection cycle is shorter compared to dairy cattle. In these cases, the choice was made to evaluate more sires through the pre-screening of candidates based on the genomic breeding values of the young sires, with the objective of increasing the selection intensity. Then, the chosen sires are evaluated using both genomic and pedigree information (Carillier et al., 2013; Baloche et al., 2014; Larroque et al., 2014). This strategy has been implemented in France since 2015 for sheep and 2018 for goats. This pre-screening option has also been tested in oil palm trees and found to be of considerable interest for choosing the best individuals with the highest genetic value in hybrid crosses before progeny testing (Nyouma et al., 2019).

For brick C, the first interest is to use genomic information for a better choice of parents to obtain high genetic gain but also to enhance genetic variability. The key point is to choose new crosses which will have a large progeny variance. Strategies to promote the best choice of parents have been used for a long time in plant breeding [see for e.g., (Dudley, 1984)] before the availability of genomic information. Bernardo (2014) tested the adaptation of classical methods for choosing parents (classes of loci, and the usefulness criterion) with genomic information through simulation and on data from maize inbreds. The interest to use genomic information has been studied further in plant breeding, with inbred lines (Lehermeier et al., 2017; Allier et al.,2019a,b) and in animal breeding with outbred populations, with applications to dairy cattle (Santos et al., 2019; Bijma et al., 2020).

The second interest of brick C is a better management of genetic variability based on knowledge of the realized kinship between individuals. In classical selection, loss of genetic diversity is evaluated through evolution of inbreeding, estimated through pedigrees. Use of genomic relationships should be more precise than pedigree relationships. However, results are contrasted with, for example, Sonesson et al. (2012) showing that genomic control of inbreeding is more efficient in genomic selection while Henryon et al. (2019) showed that optimum contribution selection provided more genetic gain using pedigree relationships rather than genomic relationships and Meuwissen et al. (2020) evidenced that the different ways to compute genomic relationships lead to different conclusions about efficiency of using genomic relationships to manage genetic diversity. However, in French dairy goats, the joint management of genetic progress and genetic variability has been implemented since 2006, with minimization of pedigree relationships for desired genetic gains (Colleau, 2002). This method was modified to take genomic relationships into account in 2018 when GS began to be implemented in French dairy goats (Colleau et al., 2017).

Globally, this brick C does not appear to be a sufficient reason for switching to GS. It is instead a bonus to be added to the advantages of another main brick and methods are still to be developed and improved.

## Discussion

### Prerequisites for Implementing Genomic Selection?

Even if, in short term, we cannot act on its level, linkage disequilibrium (LD) is one of the parameters which determines the GS efficiency. LD is an intrinsic characteristic of populations, consequence of the evolutionary forces involved in the construction of the population. With lower LD, larger reference population and/or higher marker density (in both reference and candidates populations) will be needed to achieve interesting accuracy of genomic selection. Thus, LD will impact the cost of implementing GS and a very low LD could prevent from switching to GS.

When considering criteria we can act on, the first criterion for moving toward GS is the availability of genotyping tools. Genotyping costs have decreased substantially in recent years, but technological development and large-scale genotyping still represent large investments. Human investment is also needed to acquire and use genomic information. Finally, an efficient reference population is another key point. If large reference populations have now been established in species or breeds which are widely used and/or with high economical impact, this can be still a limiting point for many species with little market size or for small breeds in animals. Genotyping investments are affordable to different degrees depending on the market size related to the species, the structure of the breeding programs and the species organization. For example, for dairy cattle, the market size and the huge number of animals all over the world make it possible to combine resources, assemble large reference populations and achieve low prices for genotyping. Moreover, offspring testing in a classical breeding program is very expensive; thus, replacing this strategy by genotyping is of economic interest. These characteristics, together with the opportunity to strongly reduce T, enabled the switch to GS in dairy cattle very early. The switch to GS is more difficult for species with smaller market sizes, those in which selection is carried out by smaller companies, or those in which GS will not decrease the cost of breeding programs. However, as genotyping costs decrease, GS becomes accessible for an increasing number of species. A significant lever is the launch of publicly supported applied research programs for the implementation of GS. These programs often enable the genotyping of the first commercial candidates and help private breeders switch to GS.

Historically, different bricks have been preferentially chosen as arguments for switching to GS at different times: brick B was the first to be chosen because it can reduce costs very significantly. Brick A came later, and the interest in this brick has increased as genotyping cost has decreased. Finally, brick C is often cited as a very interesting option but does not appear to be important enough to convince stakeholders to switch to GS. However, we expect that this brick will be used more widely in combination with bricks A and/or B to better valorise existing genomic data. Moreover, the theoretical developments related to brick C are more recent, and this brick could therefore become more popular in the future, especially for new traits, which may exhibit low genetic variability in current populations. Globally, one brick is initially decisive in switching to GS, but all benefits of GS are combined thereafter.

### Limits of Our Study

The main difficulty of this work is that for many species, GS has generally been implemented only very recently or is about to start. Thus, it is not always possible to look back to what has occurred in the past. GS is now implemented in most animal species: dairy and beef cattle, dairy goats, fish, pigs, poultry, dairy sheep, and horse in some countries. In plants, GS has been implemented mainly in species with major economic importance. Moreover, for some species (maize, wheat, and, to some extent, poultry), selection is performed by private companies, and information on their breeding strategies is thus limited.

At the beginning of our study, we thought that reviewing the literature and talking with experts would be sufficient to identify the main reason motivating the breeders of a given species to switch from a conventional breeding scheme to a breeding scheme incorporating GS. However, most papers in this field are highly prospective, exploring all possible advantages of GS without ranking them. The other available papers report simulation studies that show the efficiency of different strategies but do not truly discuss about real implementation. Finally, in some species (trees, maize, wheat, and forage plants), different strategies are relevant, and no single option presents a clear advantage over the others. For example, in maize, Windhausen et al. (2012) proposed a decrease in phenotyping effort (brick B), while Lehermeier et al. (2017) demonstrated the interest in brick C, and experts have reported that at least in one private company, brick A is implemented through a decrease in phenotyping effort (fewer repetitions and fewer environments). In these species, it is possible that different private companies have not chosen the same option for implementing GS.

### Consequences of the Biological Specificities/Breeding Organization

The specificities of the general organization of the breeding for each species has been synthetized in section “Biological and Breeding Organization Specificities.” We discuss here the consequences of these specificities on the interest for switching to GS and the strategy for implementing GS.

Switching to GS occurs more easily in contexts where the phenotyping of one candidate is rather costly, so that GS enables to make some savings on this phenotyping, at least for some of the candidates. The dairy cattle example is a good illustration of this property.

Considering the species in which GS has already been implemented, the interest for brick A vs B can be related to biological or breeding organization specificities. For example, among the species in which brick A is implemented, most are characterized by a moderate phenotyping cost and a rather short selection cycle, while most of the species in which brick B is implemented are characterized by a long selection cycle and, thus, a high unit cost of candidates. However, these characteristics are not sufficient to predict the brick chosen for GS. For example, in forest and fruit trees, at first glance, brick B seems to be a good choice because of the long duration of the breeding cycle (Kumar et al., 2012; Biscarini et al., 2017; Grattapaglia, 2017). However, due to the late age of sexual maturity in forest trees, brick B can have a limited impact on the duration of the breeding cycle. In the meantime, the implementation of brick A through genotyping associated with the integration of new selection criteria linked to traits that are difficult and costly to phenotype (biotic and abiotic resistance, for instance) could be of first interest (Lenz et al., 2020).

Thus, there is no universal key to choose the initial brick. This choice is determined by the whole context, including biological specificities, breeding organization and the economic context (commercial organization and regulations).

### Need for More Generic Studies of GS Efficiency

A large part of the literature on the analysis of the reasons for implementing GS is limited to one species or even to a population or a limited set of closely related species/populations. These specific investigations are necessary to define the best GS strategy for each context. However, transversal analysis, such has the one proposed here can be of interest in at least two situations. A first situation corresponds to the case where, for a given species, very few publications and research has been made for implementing GS for this species. In such a case, the breeder may be interested in what we can learn from other species or populations. This paper can provide him/her a synthetic overview of the options that have been studied or implemented in other species. A second situation corresponds to a case where a reader is interested to have an overview of the possible applications of GS, rather than the application of GS in one particular species or population. This can be the case for example with scholars from other disciplines like social science. This can also help explaining the options for implementing genomic selection, in rather simple terms, to a general audience (e.g., undergraduate students and decision makers not directly involved in breeding).

We show here that the arguments for implementing GS are generic and may be applied to a wide range of species. More precisely, we show that a very limited set of basic common bricks can be used to summarize the reason for implementing GS in a wide range of animal and plant species. We think that transversal analysis, comparing a given strategy across very different contexts (biological specificities and breeding organization), should be extended. We expect, in the coming years, to have more evidence on the actual implementation of GS for a larger range of species and the impact of GS. If this is the case, the current analysis could be updated and probably be made more rigorously, with a quantitative analysis of the literature. Of course, in the end, stakeholders will make their own choice, which will be specific to each context, but such transverse analysis should help to anticipate the advantages and drawbacks of different strategies.

## Data Availability Statement

The original contributions presented in the study are included in the article/supplementary material, further inquiries can be directed to the corresponding author.

## Author Contributions

R2D2 members provided the relevant literature, participated in discussions to compare the breeding programs in different species, and more precisely defined the bricks and their interest related to different species. SL proposed the original idea of bricks. AF-S performed the bibliographic analysis of the reviewed papers. CB, AF-S, MD-N, and SL analysed all the information (e.g., manuscripts and discussions). MD-N, SL, and AF-S wrote the final manuscript. All authors read and approved the final manuscript.

## Conflict of Interest

The authors declare that the research was conducted in the absence of any commercial or financial relationships that could be construed as a potential conflict of interest.

## Footnotes

1 https://colloque.inrae.fr/metaprograms-workshops_eng/Metaprograms/Selgen
